# Supplementary material for: hu.MAP 2.0: integration of over 15,000 proteomic experiments builds a global compendium of human multiprotein assemblies
Source: Mol Syst Biol. 2021 May 11;17(5):e10016. doi: 10.15252/msb.202010016 (PMC8111494; doi:10.15252/msb.202010016)
Supplement: Supplementary file 1 — Expanded View Figures PDF [file MSB-17-e10016-s002.pdf]

Expanded View Figures

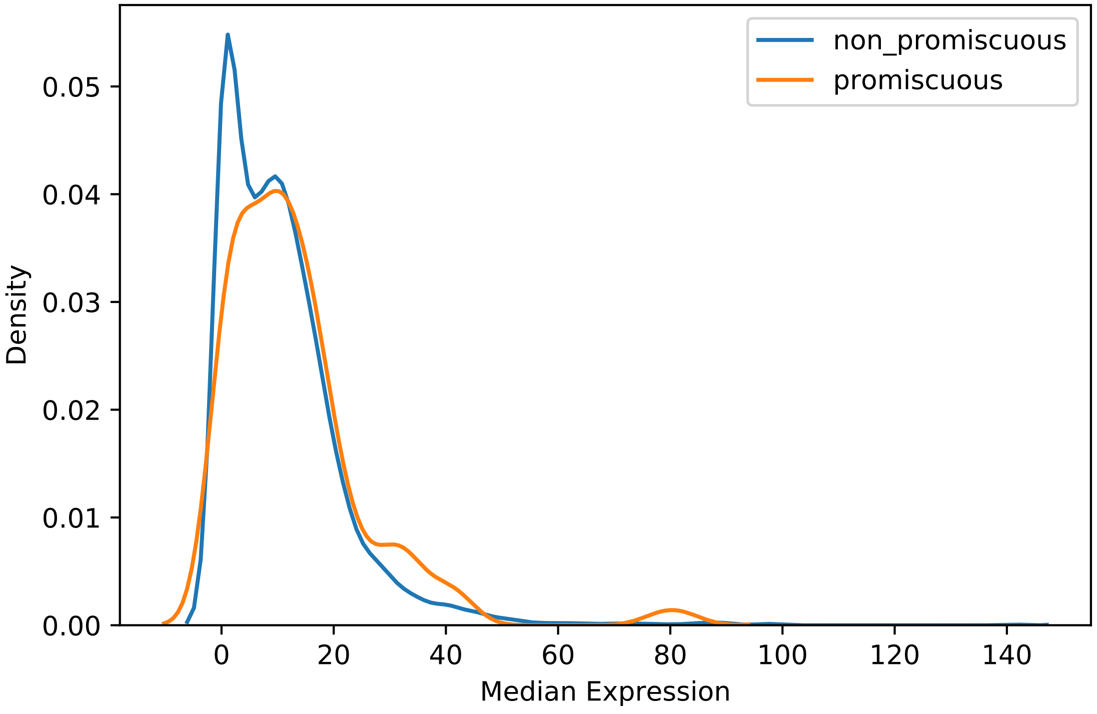

Figure EV1. Density plot of Human Protein Atlas (Uhlén *et al*, 2015) transcript expression across tissues of promiscuous proteins versus non-promiscuous proteins.

We observe negligible differences between promiscuous and non-promiscuous distributions suggesting expression levels are not a factor contributing to the identification of promiscuous proteins.

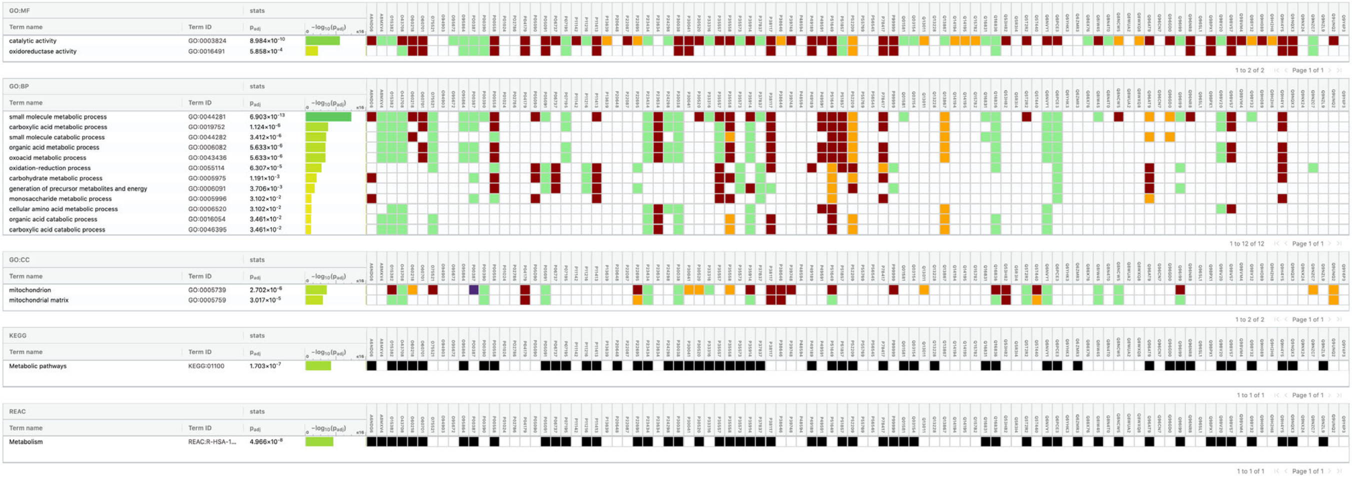

Figure EV2. Annotation enrichment of older promiscuous proteins.

gProfiler output shows older promiscuous proteins are enriched for metabolic processing annotations.

© 2021 The Authors

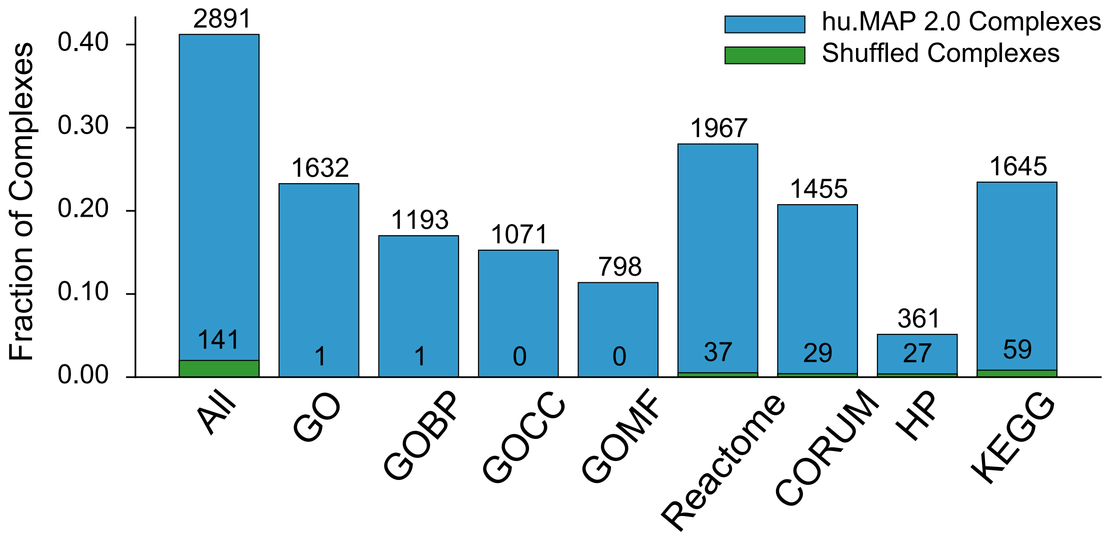

Figure EV3. hu.MAP 2.0 complexes are functionally enriched.

The bar chart shows the number of identified complexes that are enriched with at least one annotation from GO, Reactome, CORUM, KEGG, or Human Phenotype Ontology (HP) at an FDR threshold of 0.05.

Figure EV4. SVM confidence score versus test set precision.

The line plot shows the relationship between the SVM confidence score, and the empirical precision value calculated from the test set of protein interactions. The relationship shows the precision value is consistently higher than the confidence score.

 © 2021 The Authors

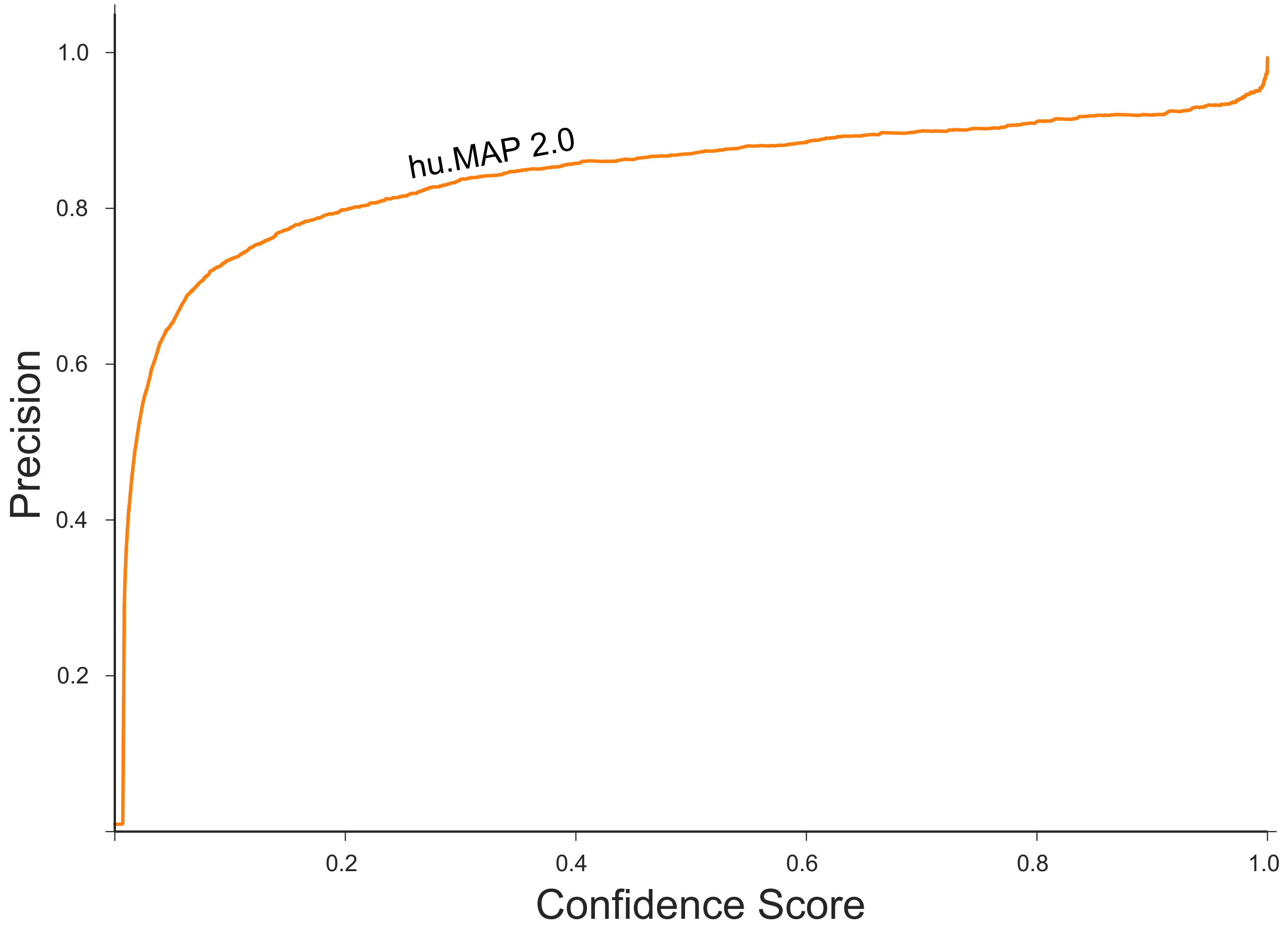
hu.MAP 2.0
Precision
1.0
0.8
0.6
0.4
0.2
0.2
0.4
0.6
0.8
1.0
Confidence Score

Figure EV4.
